# Control of Antimicrobial Resistance Requires an Ethical Approach

**DOI:** 10.3389/fmicb.2017.02124

**Published:** 2017-11-02

**Authors:** Ben Parsonage, Philip K. Hagglund, Lloyd Keogh, Nick Wheelhouse, Richard E. Brown, Stephanie J. Dancer

**Affiliations:** ^1^Department of Engineering, University of Strathclyde, Glasgow, United Kingdom; ^2^Department of Engineering, Luleå University of Technology, Luleå, Sweden; ^3^School of Applied Sciences, Edinburgh Napier University, Edinburgh, United Kingdom; ^4^Sophrodyne Ltd., Glasgow, United Kingdom; ^5^Department of Microbiology, Hairmyres Hospital, NHS Lanarkshire, Bothwell, United Kingdom

**Keywords:** ethics, antimicrobial resistance, antimicrobial stewardship

## Abstract

Ethical behavior encompasses actions that benefit both self and society. This means that tackling antimicrobial resistance (AMR) becomes an ethical obligation, because the prospect of declining anti-infectives affects everyone. Without preventive action, loss of drugs that have saved lives over the past century, will condemn ourselves, people we know, and people we don’t know, to unacceptable risk of untreatable infection. Policies aimed at extending antimicrobial life should be considered within an ethical framework, in order to balance the choice, range, and quality of drugs against stewardship activities. Conserving availability and effectiveness for future use should not compromise today’s patients. Practices such as antimicrobial prophylaxis for healthy people ‘at risk’ should receive full debate. There are additional ethical considerations for AMR involving veterinary care, agriculture, and relevant bio-industries. Restrictions for farmers potentially threaten the quality and quantity of food production with economic consequences. Antibiotics for companion animals do not necessarily spare those used for humans. While low-income countries cannot afford much-needed drugs, pharmaceutical companies are reluctant to develop novel agents for short-term return only. Public demand encourages over-the-counter, internet, black market, and counterfeit drugs, all of which compromise international control. Prescribers themselves require educational support to balance therapeutic choice against collateral damage to both body and environment. Predicted mortality due to AMR provides justification for international co-operation, commitment and investment to support surveillance and stewardship along with development of novel antimicrobial drugs. Ethical arguments for, and against, control of antimicrobial resistance strategies are presented and discussed in this review.

## Introduction

Antimicrobial agents play a huge role in medical practice. The facility for healthcare services to reliably cure the infected patient has saved countless lives over the last century. Since the discovery of penicillin by Alexander Fleming in 1928, antibiotics have revolutionized patient management, with the 1950–1970’s known as the “golden era of antibiotics.” Medicine reached a pivotal point in the 1960’s with the introduction of multiple agents, which could seemingly treat any infection. This received due comment from then US Surgeon General who infamously declared that the threat from infection had been relegated to the past (Infectious Diseases Society of America [IDSA] et al., 2011). Nowadays, increasing resistance is seen as a major problem, since there have been diminishing numbers of novel antibiotics approved since the late 1970’s (Infectious Diseases Society of America [IDSA] et al., 2011). It appears that the microbial propensity to develop resistance is occurring much faster than man’s ability to develop new agents.

One of the first warnings of antimicrobial resistance (AMR) came from Fleming himself during his Nobel Lecture in 1945, even though antimicrobial therapy was a relatively new concept. He said:

“… I would like to sound one note of warning. Penicillin is to all intents and purposes non-poisonous so there is no need to worry about giving an overdose and poisoning the patient. There may be a danger, though, in under dosage. It is not difficult to make microbes resistant to penicillin in the laboratory by exposing them to concentrations not sufficient to kill them, and the same thing has occasionally happened in the body. The time may come when penicillin can be bought by anyone in the shops. Then there is the danger that the ignorant man may easily under dose himself and by exposing his microbes to non-lethal quantities of the drug make them resistant. Here is a hypothetical illustration. Mr. X. has a sore throat. He buys some penicillin and gives himself, not enough to kill the streptococci but enough to educate them to resist penicillin. He then infects his wife. Mrs. X gets pneumonia and is treated with penicillin. As the streptococci are now resistant to penicillin the treatment fails. Mrs. X dies. Who is primarily responsible for Mrs. X’s death? Why Mr. X whose negligent use of penicillin changed the nature of the microbe. There is a moral here, and that is that if you use penicillin, use enough” (Fleming, 1945).

Fleming was correct, since this story illustrates an important point about appropriate usage of antibiotics, but escalating AMR deserves a much broader approach than appealing to the moral principles of an individual. Antimicrobial agents are unique because they are the only pharmaceutical drugs that have transmissible loss of efficacy over time (Spellberg et al., 2016). The world is fast approaching a time whereby people will die from infections because there are no longer any effective drugs (Gallagher, 2015).

This will change the face of medicine, as we know it, since many modern medical procedures are highly dependent upon antimicrobial protection. This might appear to be chiefly a problem for doctors and scientists but the nature of widespread AMR embodies a large number of ethical and moral issues, influencing every aspect of infection management and potentially affecting everyone. This review aims to introduce and discuss some of the most important ethical dilemmas associated with AMR and its control, including national and international responsibilities, pharmaceutical companies, prescribing, education, advertising, veterinary and agricultural practices, infection control, and patient behavior.

## Why Antimicrobial Resistance Is An Ethical Problem

The moral implications of an action or non-action constitute the need for ethical consideration. This encompasses prevention and control of AMR (Littmann et al., 2015). The risks from AMR are clear – untreatable infection – and these risks are well-established. With this knowledge comes the responsibility to act, in order to slow resistance trends in order to provide time to find new ways of treating infection. If nothing is done, then future patients will be deprived of potentially lifesaving drugs. This is unacceptable, especially after humanity has experienced a ‘golden era of antibiotics.’

Who is accountable for action? There are both individual and institutional responsibilities toward control of AMR. These include healthcare prescribers, their practices and choice of antimicrobial drug; educational organizations that establish the qualifications required to prescribe anti-infectives; pharmaceutical companies who develop and market antimicrobial products; drug discovery scientists and/or the academic institutions that support them; veterinary and agricultural industries; ordinary people who request, buy, take (or fail to take) antibiotics; and executives responsible for health regions, or indeed, national bodies including current political rulers. These will be discussed further, but the responsibility clearly encompasses both governmental and professional institutions as well as prescribers and their patients.

Whatever actions are chosen to redress AMR, a balance is required between the needs of the individual and the common good. For example, prescribers may strive to restrict drug consumption, but benefits from decreasing AMR for future use might condemn current patients to serious sepsis. Who should receive antimicrobial agents and who should not? What criteria are required to release or withhold curative or even potential lifesaving drugs? If there is a choice between efficacy and risk of resistance, what constitutes appropriate *and* prudent prescribing? There are additional ethical dilemmas involving antimicrobial prescribing for subsets of patients with specific conditions, none more pertinent than prescribing powerful drugs to terminal or elderly patients (Leibovici and Paul, 2015). Indeed, antimicrobials used to reduce the risk of infection for some (healthy) patient populations should be curtailed, given that these patients are generally well (Haire and Kaldor, 2013). While physicians have an obligation to help their patients, they are expected to refrain from causing harm (Gillon, 1994). As with all prescribing, a fundamental principle of beneficence may be illustrated by the balance between therapeutic benefit and adverse effects from the drugs prescribed. More pertinent to antimicrobial prescribing, the term non-maleficence meaning ‘to do no harm’ or rather inflicting the least harm possible to reach a beneficial outcome, defines the choice between immediate benefit from anti-infectives and potential lack of therapies for that patient in the future. This term could also theoretically be applied to current practice of giving antibiotics as surgical prophylaxis, perhaps a privilege in the future (Dancer, 2013b).

Prescribing restrictions challenge traditional autonomy of individual practitioners. Autonomy itself demonstrates an ethical principle intimating freedom from external control or influence. In this respect, doctors should prescribe what they believe to be best for a patient, justifying empirical decisions with clinical assessment, experience, and sometimes psychosocial reasons (Dancer, 2004; Spellberg et al., 2016). Attempts at influencing medical autonomy means that doctors may not support restrictive policies, particularly if they feel that these short-change a particular patient or condition, or if cost considerations determine therapeutic choice. There remains a fiduciary responsibility to individual patients with shared decision-making balanced against long-term societal interests. Patients themselves have needs, values, and preferences, and their wishes provide a different type of challenge. Directing the medical profession over their right to prescribe requires sensitive diplomacy, underpinned by evidence-based science. Opposing arguments include internet and over-the-counter sales of antimicrobials, which circumvent the role of licensed prescribing. Antibiotics are a shared global resource and clinicians, patients, public health, and government are bound together by the need to protect these drugs from misuse (Spellberg et al., 2016).

Mandated restrictions may encompass use of human agents in veterinary and agricultural practices. Growth promotion in animals has been banned in the European Union but not necessarily elsewhere. Aside from food-producing animals, the public expect antimicrobial drugs for sick pets and indeed, wildlife. Antimicrobials are also widely used in business and industry, with products incorporated into paints, sprays, coatings, fabrics, and plastics. Antiseptics, now definitively linked with antibiotic resistance, are present in a huge array of household items (Dancer, 2013b). Thus, commerce and profit provide universal challenges to international bodies supporting stewardship.

There are further ethical considerations over the distribution of antimicrobial agents. Should drugs be limited to people based on residence in a particular healthcare facility; district; region; or even country (Littmann et al., 2015)? Who decides whether some patients should forfeit medicines, even though that decision may conflict with beneficence and indeed, all the fundamental principles of medical ethics? To a certain extent, this is happening already, due to high pricing of specific drugs and countries facing sanctions, wars, economic disadvantages and/or lack of public health structures. The spread of multi-drug resistant tuberculosis (MDRTB) through populations riddled by HIV infection provides a good example of the latter. Since drug resistance is not confined to one region of the world, antimicrobial provision and management should generate discussion and policy on a global basis (Dancer, 2013b).

Antimicrobial resistance raises further economic issues, since increasingly resistant infections encourage clinical demand for more spending on remaining drugs, forcing governing bodies to consider selective funding. Expensive last-resort antibiotics may serve only to accelerate further resistance and could challenge supplier strategies (Littmann and Viens, 2015). Indeed, private medicine consortia may decide to act independently of any governmental decree. Universal access to effective antibiotics is essential for tackling antibiotic resistance (Daulaire et al., 2015). A related issue operates at local level, whereby some institutions advise prescribers to choose cheaper options, even if these are therapeutically suspect. Such practices are hardly judicial, since they enhance the risk of persistent or recurrent infection, which itself encourages resistance.

Controlling AMR is an on-going problem with multiple ethical issues, with no possible single solution that does not raise even more questions. The varied strands of medical practice and autonomy, prescribing, access to drugs and economic status versus increasing resistance lend themselves to ethical interpretation at every level. The continuing process of combating resistance requires much debate regarding the implementation of global initiatives (Littmann and Viens, 2015). Restricting antibiotics to benefit one stakeholder will almost certainly affect the same or a different stakeholder further down the line (Dancer, 2004).

## Perspectives on Antimicrobial Stewardship

### Hospitals

Antimicrobial stewardship aims to prevent unnecessary use of drugs within healthcare (Dellit et al., 2007). Careful stewardship also protects patients from adverse effects or reactions with other drugs. This is approached as a predominantly scientific problem in most hospitals, with guidelines on antibiotic choices balanced against diagnosis, concurrent therapy, toxicity, allergies, and length of course. A senior practitioner leads an antimicrobial committee, supported by clinical microbiologists, General Practitioners (GPs), infectious disease specialists and pharmacists. For prescribers, decisions must be made over when patients should receive antibiotics and which class or combination would be most beneficial. Both antimicrobial committee, with agreed policies, and prescribers, with therapeutic choices, need to champion the collective good over individual rights. The final decision is a trade-off between treating a sick patient versus the admittedly nebulous effect of prolonging therapeutic effectiveness for the future (Littmann and Viens, 2015). Prescribers can witness the impact of chosen therapy according to patient response in the short term, but they cannot necessarily see longer-term benefits on the local environment emanating from careful stewardship (Dancer, 2004; Dancer et al., 2006).

How far should a committee insist on adhesion to agreed guidelines? Is it appropriate to ‘ban’ certain agents, while championing others, especially if the latter are less effective? Mandating guidelines into policy runs the risk of alienating prescribers, however, well-intentioned. Some cases cannot be managed using generalized guidelines. This is due to individual patient characteristics or infection type, and/or antibiotic susceptibility profile of the infecting pathogen(s). There may also be institutional factors, with an on-going outbreak or threat of one, which skews choice of therapy. Thus, guidance should only be that; advisory rather than mandatory. Imposition of prescribing penalties, while generally unethical, could conceivably challenge diagnostic skills and experience, as well as promote defensive behavior by prescribers. If a doctor knows he will not be penalized for denying antibiotics for a particular patient, even if the infection risk escalates for that patient, then the decision to withhold therapy becomes easier. A ‘wait and see’ policy is clinically acceptable provided the patient remains systemically well. Conversely, a potential legal challenge could threaten balanced decision making, particularly in private practice situations. Mandatory policies are also at risk of censorship due to economic, rather than efficacy considerations, which provides further ethical bias.

Stewardship would be supported better by improving the current range and provision of infection screening processes (Perez et al., 2014; Daulaire et al., 2015). These deliver quicker and more accurate detection of infection and pathogen characteristics. Diagnostic microbiology is key to providing the information required for appropriate prescribing, because knowing the identity of the pathogen benefits not just the affected patient but future patients. Correct therapy reduces the risk of spread. Unfortunately, rapid microbiological tests are dependent upon available resources and infrastructure of healthcare institutions. While additional funding for expensive molecular tests requires a thoughtful business case in developed countries, poorer countries lack sufficient diagnostic laboratories. Arguments for implementing or improving diagnostic provision are dependent upon published evidence, which, in company with infection prevention strategies, is virtually non-existent. There is an ethical trade-off between investments in diagnostic facilities versus potential transmission of key resistant pathogens, quite apart from the danger of diagnostic delay for an individual patient. You cannot control what you do not know about.

Clinical expertise starts with experience, level and quality of the prescriber’s education. Up to 50% of antibiotics used in United States hospitals are inappropriate, which questions the educational curriculum underpinning medical training (Centers for Disease Control and Prevention [CDC], 2014). If a student does not receive basic education on microbiology, infection control and appropriate prescribing, or understand why these are important, then future stewardship initiatives will not achieve ownership (Owens et al., 2009). This could be ameliorated by introducing structured feedback on prescribing, which influences adaptive behavior (McLellan et al., 2016). Alternatively, senior doctors could take more responsibility for antimicrobial choice. This could be formalized by health boards or even national bodies, since expecting seniors to oversee antimicrobial choice and consumption for their patients cannot be disputed on ethical grounds. Prescribing is often left to junior doctors, who may not have the experience to deal with complex cases.

Hospital managers report considerable structural and inter-professional challenges to implementing antibiotic optimisation and governance (Broom et al., 2016). Good prescribing is given lower priority vis-à-vis other managerial issues in hospitals. Why is this? Perhaps because there are few, if any, immediate effects from poor choice of antimicrobial agents. Patients will get better, or not, despite inappropriate prescribing. Secondly, there may be antipathy between clinicians and those in managerial positions, each misunderstanding what they do, with inevitable loss of respect in both directions. Further, management-directed changes are constrained by the perceived absence of a “culture of accountability” for antimicrobial use amongst doctors. It is clearly unethical for non-clinical managers to manipulate prescribing in order to support a stewardship strategy but they may feel that there is no alternative without clinical engagement. All clinical and managerial leaders should become, and remain, committed to controlling AMR in order to understand and address the barriers to change. Better relationships between clinicians and managers would help to embed a stewardship program within the healthcare framework.

### Community

Prescribers in community-based practices are less well-supported than hospital colleagues, since they often operate in isolation. Despite a plethora of prescribing guidance, there are many opportunities for incorrect choice of anti-infectives as well as over or defensive prescribing. Patients not only desire the best and most modern of treatments, they expect a ‘pill for every ill.’ This makes it difficult to withhold antimicrobial drugs from the worried well, especially those who are paying for their treatment. Doctors are more likely to prescribe drugs for private patients, because they feel obliged to do so (Duane et al., 2016). There may well be an ethical responsibility to challenge prescribers over their management of private vs. public patients, while acknowledging the tension between patient autonomy and stewardship requirements.

The most pertinent threat to stewardship in community clinics is time constraints. A packed waiting room will not permit the explanation, reassurance and education required for non-infected patients, especially parents who want something for a sick child (Verheij, 2009). An important but largely unstated issue in ethical guidance is to consider any possible alternatives, or what the trade-offs from different choices may be; thus, there are such arguments for and against stewardship initiatives. Without time to discuss options, GPs are placed at risk of prescribing in order to move the clinic along. Indeed, rigid stewardship occasionally compromises the management of a patient who really does need therapy (Kieran et al., 2011). This is compounded by poor, or delayed, access to diagnostic laboratories, which encourages clinicians to prescribe broader-spectrum agents, ultimately accelerating AMR (Dancer et al., 2015). A doctor is at risk of prescribing antibiotics ‘just in case’ if the patient is a child, or vulnerable in some way. This obviously compromises guidance on prescribing, although it might protect the doctor from future legal challenge in the event of complications. Defensive prescribing further strengthens the need to support doctors’ decisions on anti-infectives (Duane et al., 2016).

Like all doctors, GPs expect autonomy in their prescribing choice; authorities challenging this require robust evidence and recognized professional standing (Dancer, 2016). It is possible that rigid adherence to medical beneficence fails to recognize the risk to future patients from antimicrobial prescribing. Most doctors are affiliated to a national professional body that oversees practice in their specialty. There are also national institutions that regulate doctors’ services and behavior, such as the United Kingdom General Medical Council (GMC). While these organizations have ethical responsibility to define and support antimicrobial stewardship, there have not been any mandates issued on antimicrobial prescribing. Should restrictions be imposed, then doctors will require education, audit, and leadership from national bodies. These should surmount all the barriers to stewardship already mentioned but may still fail when challenged by workload priorities. There is increasing interest in applying behavioral science for doctor-led prescribing (Duane et al., 2016; McLellan et al., 2016). Social norm feedback to prescribers can be an effective method to reduce total antibiotic consumption in ambulatory care, despite lack of evidence on outcomes such as resistance, appropriateness of antibiotic use or harm from underuse (Gould and Lawes, 2016; Hallsworth et al., 2016).

Are there ethical considerations toward allowing patients to prescribe their own drugs? This might alleviate delays in treatment, but there will always be a risk that the patient chooses ineffective medication, or dose, which fails to treat the original infection, causes adverse affects and encourages resistance. For well-informed patients with chronic conditions, choice of therapy can be regarded as ethically acceptable, provided they do not change or store the drugs or donate to others. Offering a prescription following initial consultation and asking the patient to make a decision on a wait-and-see basis is tried and tested, with some success (Little et al., 2014). Given support and informed advice, patients themselves deserve some degree of autonomy, or empowerment, toward their clinical management (Gillon, 1994).

Rigid stewardship, including prescriber penalties, might be seen as inappropriate when undermined by private practitioners, black market, counterfeit, over-the-counter and internet drugs (Delepierre et al., 2012). The practice of buying antibiotics from a shop or online, without any form of prescription, promotes AMR because it might not be the correct drug or dose for the user (Kobaidze et al., 2009). This illustrates an unknown and unregulated issue for global control of AMR. Public purchase of antimicrobial drugs challenges prescribing rights and freedom of choice for doctors when people can obtain so-called restricted drugs with minimal effort (McKenna, 2015). Despite this, doctors who prescribe antibiotics online are currently at risk from GMC investigation (Torjesen, 2016). Penalties for poor prescribing choices could well be justified, if the objective is to spare future patients from multi-drug resistant pathogens. This would apply to telephone consultations as well as internet-based prescribing.

A 2014 survey conducted by the World Health Organization (WHO) reported that of 43 European countries, 19 allowed certain antibiotics to be purchased from pharmacies (WHO, 2014). The report concluded that pharmacists could do more to restrict and improve antibiotic use for the public. Too many people misunderstand the principles underlying stewardship, which means that they do not finish the prescribed course, demand more or alternative agents, give drugs to others, or even stockpile agents at home (Zoorob et al., 2016). Countries in which antibiotics can be obtained without prescription should consider regulating the distribution of antimicrobial agents. Restrictions such as official prescription, or auditing antibiotics purchased online, would help national stewardship policies. If unregulated access could be accurately monitored, then it would be easier to target outlets of non-prescribed antimicrobial drugs, including counterfeit agents, with intention toward international control.

### Prescribing Guidelines

There are varying and contradictory differences between antimicrobial guidelines, which tend to be couched in technical terms and aimed at professionals such as doctors, scientists, and pharmacists (Dellit et al., 2007). This effectively makes them “inaccessible” to the layman and means that patients may not understand, or know about their existence, despite the fact that they themselves are directly affected by the contents (Micallef et al., 2017). Non-expert stakeholders might well have a perspective that would challenge cost issues and alternatives. Indeed, is it morally acceptable to prescribe an agent to a patient in order to comply with local regulations, when there is another drug that might be more effective, or quicker, or with fewer adverse affects? Discussion over choice, while balancing both medical and patient autonomy, requires time during a consultation; time, which the prescriber may not have. It is also possible that the prescriber will not necessarily know the full range of options and has to rely upon personal beliefs and experience, as well as any guidelines at hand.

Most guidelines focus on hospitals, rather than community, since the risk of generating resistance is assumed to be greater in environments that are heavily exposed to antimicrobials. GP prescribing is not thought to constitute the same degree of risk as a hospital patient receiving powerful parenteral agents. This may erode support for stewardship principles in the community. Furthermore, the long-term effects of over or inappropriate prescribing in the community are far more difficult to monitor than for hospital environments. Each prescription, community or hospital, will ultimately exert an effect on the local environment, whether measurable or not. Repeated antibiotic courses for community-based patients has been shown to precipitate hospital admission due to multi-drug resistant organisms (Hillier et al., 2007; Costelloe et al., 2010).

Guidelines might exhort prescribers to prescribe more carefully but these are not mandatory policy (Duane et al., 2016). Doctors can still prescribe their preferred drug without recourse, unless they can be held accountable for their choice. The quality of guidance itself is important, since conflicting advice provides justification for using ‘favorite’ antibiotics. Restrictive guidelines do not necessarily guarantee the correct choice of drug. Prescribers may also know of previous resistance demonstrated by a particular patient or locally circulating pathogen and use this to circumvent guidelines (Duane et al., 2016). While there may be a strong moral argument to choose unusual or second-line drugs, a prescriber could be placed at risk of penalty unless relevant and timely confirmation is received from the diagnostic laboratory. This demands a degree of courage (and experience) among prescribers, as well as the necessary diagnostic support. There is good evidence to show that restrictive and enabling interventions empower doctors to both reduce and improve antimicrobial prescribing, although the most effective behavioral techniques do not yet seem to be widely implemented (Rawson et al., 2017).

### Agricultural and Veterinary Use of Antibiotics

Half of the world’s consumption of antibiotics is used for animals and agriculture. Livestock use is predicted to increase by two-thirds between 2010 and 2030 years (Van Boeckel et al., 2002). Whether used for growth promotion, therapy or prophylaxis, dosing animals with antibiotics encourages worldwide AMR (Van den Bogaard et al., 2001; Smith et al., 2002). Use of antimicrobial agents for anything other than direct treatment of infection should now be regarded as unethical, unless the agents chosen are excluded from the human formulary. If bacteria develop resistance within a food animal, then resistant strains spread throughout the farm, colonize other livestock and persist in the environment. Newly procured stock is at risk of acquiring organisms from the local environment unless the farm practices all-in all-out management or effective hygiene for contained pens. Farmers and their families are also at risk of acquiring resistant organisms from the animals for which they care (Levy et al., 1976; Van den Bogaard et al., 2001). Multi-resistant bacteria then pass along the food production chain to consumers via abattoirs, appropriately termed ‘farm-to-fork’ (Warren et al., 2008). Control interventions for AMR are thus traded for food security. This illustrates an ethical impasse, which asks whether untreatable infection, or food shortages, constitute the greater population risk. Traditional bioethical principles cannot answer this question easily, given that the issues are tempered by economic status, public health, and pure luck.

Most antimicrobial consumption in agriculture is aimed at growth promotion or infection prevention, rather than direct treatment of infection, and this obviously raises ethical issues. There are economic reasons for livestock workers to use antimicrobials because production benefits could be minimal even within optimized systems. However, controlling consumption may be achieved with minimal losses in animal performance. Examples from Denmark and the Netherlands illustrate the effects of government intervention and legislation on decreasing antimicrobial use while at the same time increasing animal production. Denmark began to reduce antibiotic growth promotion in 1995, eventually banning the practice in 2000 (The, 2012). In the Netherlands, a ban was not introduced until 2006, when the EU-wide ban was implemented (Speksnijder et al., 2015). Both countries saw initial increases in therapeutic AMR consumption, possibly from reduced prophylaxis but also due to ambiguities in the terminology of therapeutic and non-therapeutic use. Danish pig production increased by 47% between 1992 and 2008, with antimicrobial use decreasing by 51%; antimicrobial use in poultry reduced by 90% between 1995 and 2008 without apparent loss in production. In the Netherlands, antimicrobial use reduced by 56% between 2007 and 2012 without production losses. The Dutch experience suggests that farmers shifted focus from using antimicrobials toward better management systems, which circumvents ethical arguments over banning antibiotics for growth promotion. The number of Danish producers has indeed declined, suggesting that only farms with good practices have been able to maintain profitability since the ban (The, 2012). Clearly, well-resourced countries have been able to reduce total antimicrobial consumption without affecting animal production. Similar restrictions in low-income countries, however, may compromise suboptimum farming systems. Despite support for a global blanket ban on growth promotion and prophylaxis, there are arguments for equivalent need to protect food production in poorer countries (Littmann et al., 2015). This supplies a different view toward the same argument regarding well-resourced countries.

Therapeutic use of antimicrobial agents in veterinary medicine is another important issue (Catry et al., 2010). As with people, treating animals presents similar moral issues, but with the added responsibility of balancing animal health against potential long-term effects of AMR for humans. One of the key ethical dilemmas in this regard is the continued use of colistin for livestock. Colistin has been used since the 1950’s to treat a variety of conditions caused by Gram-negative bacilli in various livestock species, but most commonly for digestive disorders in pigs. For much of this period, use of colistin in humans has been restricted toward topical use and pre-operative bowel sterilization due to its systemic toxicity. Recently, however, colistin has become a last-line antimicrobial in human medicine for the treatment of infections caused by carbapenemase-producing bacteria. While it may not be possible to curtail use of colistin in veterinary medicine, perhaps measure should be imposed to restrict the drug for treatment rather than prophylaxis (Catry et al., 2015; Rhouma et al., 2016). In pig production, 99% of colistin use is for mass oral administration (European Medicines Agency, 2016), i.e., metaphylactic use, whereby groups of animals, both sick and healthy, are treated at the same time (Trauffler et al., 2014). The fact that such a precious drug of last resort is given to healthy livestock clearly contravenes a range of judicial arguments. However, a move toward targeted treatment would require a significant change in the mode of administration for livestock owners, as well as enhanced financial penalty with the need to identify, isolate and treat individual animals.

Antibiotics for human use should not be jeopardized by inappropriate prescribing for companion animals or livestock (Collignon et al., 2009; FDA, 2012). As with banning growth promotion, commitment to change may only occur through mandatory restrictions, surveillance and testing in veterinary practices and agriculture, including imported livestock and foods. Ethical reasoning becomes less persuasive when challenged by humane support for sick pets or livestock profits. Food producers and pet owners can, if wanted, bypass local prescribing restrictions and purchase what they want from internet or other sources. Since their choice may be inappropriate, there is an argument for flexibility in national guidelines, rather than a total ban for animals.

## Pharmaceutical and Regulatory Authorities

Pharmaceutical companies are reluctant to pursue anti-infective development for largely economic reasons (Brogan and Mossialos, 2013). Developing new drugs is a long and costly process, without even the certainty of useable results (DiMasi et al., 2004). Antibiotics are generally used for a few days, unlike drugs aimed at chronic disease. Even if a new drug gains approval, healthcare systems may reserve it as a last resort, in case resistance develops. It is even possible that potential target sites for engineering antimicrobial activity have already been squandered (Cormican and Vellinga, 2012). Thus, pharmaceutical consortia are putting forward fewer resources toward research to develop novel agents and vaccines. The number of approved antibiotics within the United States from 1983 to 2012 continues to decline (Infectious Diseases Society of America [IDSA] et al., 2011).

What would happen if drug companies increased the price of antibiotics in order to pursue development of new agents? This would force government and healthcare systems to focus on antibiotic consumption, although there is no guarantee that surplus income would be directed toward anti-infective development. Price increases would create serious ethical problems for poorer countries that have neither the money for expensive drugs, nor the means to allocate or distribute effective antibiotics (Laxminarayan et al., 2016). Inflated costs would serve only to nullify any existing distribution system and might encourage black market trade of counterfeit or time-expired antibiotics. People would attempt to treat themselves, with risk of chronic illness or death. A judicial government should surely not permit a population to treat themselves. Inappropriate choice of drugs would impact both on individual and society, as well as encourage resistance; the latter amplified in environments lacking sanitation or clean water. There is a clear ethical obligation for wealthier countries to front the cost of drugs and vaccines, so that less well-resourced countries need contribute only a nominal fee (Brogan and Mossialos, 2013).

While regulatory barriers to antibiotic innovation are frequently blamed for stalling antimicrobials, truly novel antibiotics are not often shelved due to such barriers (So and Shah, 2014). When compared with other drugs, antimicrobial agents have both a higher success rate of US Food and Drug Administration (FDA) drug approval and a shorter approval time, although it is true to say that recent drugs have usually evolved from pre-existing antimicrobial classes. Different regulatory tiers of approval exist; depending upon the type of trials performed and perceived unmet (urgent) need for specific agents. In 2010, the FDA issued draft guidance calling for scientific justification of margins in non-inferiority trials for treating acute bacterial skin infections (FDA US Center for Drug Evaluation and Research, 2010). This is where an established drug is compared against a more recent version for a specific indication, in this case, skin infection, but without the facility to measure any new benefits (Outterson et al., 2013). While the need for anti-infective approval is obvious, there are ethical challenges for strategies aimed at accelerating such approval. There is a risk that so-called non-inferiority trials might rely upon poorly defined or unreliable outcome criteria; they certainly should not replace superiority trials, usually involving a new antibiotic class, and therefore potentially more valuable.

Toxicity issues will often terminate the life of an antibiotic. Of around 60 antibiotics approved as new entities between 1980 and 2009, 43% were withdrawn as compared with 13% among non-antibiotics (Outterson et al., 2013). The approval pathway of telithromycin, a first-in-class ketolide antibiotic, is a case in point. There were reports of severe liver injury linked with this drug, which heralded urgent safety warnings and congressional investigations into the FDA’s acceptance of fraudulent safety data and trial methods (Ross, 2007). The FDA subsequently concluded that non-inferiority trials, then considered appropriate for telithromycin, were no longer acceptable. There were also concerns over bedaquiline, another first-in-class drug for multidrug-resistant tuberculosis. Bedaquiline received accelerated approval on the basis of a single phase II clinical trial despite a hugely inflated mortality rate in treated subjects than for those receiving placebo (Carome and Wolfe, 2012). Accelerated approval is not the same as general FDA approval, however, since it comes with strict limitations and is aimed at providing unmet need for treatment (FDA US Food and Drug Administration, 2004). The bioethical principles demonstrated here, concern the balance between unmet need and the clinical safety of new drugs (Outterson et al., 2013). No medical practitioner wishes to invoke maleficence, even when faced with few therapeutic options. Anxiety over diminishing antimicrobial drugs should not permit trials to circumvent appropriate testing or regulatory barriers.

## Responsibilities of Governing Bodies

The United Kingdom has launched multiple activities aimed at tackling AMR, some of which involve institutions such as the World Health Organization (Chan, 2011; O’Neill, 2014). The European Union and the European Federation of Pharmaceutical Industries and Associations have created a joint project called ‘Innovative Medicines Initiative,’ which includes a program worth €700m entitled, “New drugs for bad bugs” (Innovative Medicines Initiative [IMI], 2015). The 2012 campaign covers all aspects of antimicrobial development and began as a public/private partnership intended to fund small companies to work alongside larger companies in order to focus on antibiotic research.

The United Kingdom Five Year Antimicrobial Resistance Strategy (2013–2018) was developed with devolved administrations including bodies that will be responsible for delivering the work (O’Neill, 2014). The strategy highlights the role of public awareness campaigns and the need to improve sanitation and hygiene; it also mentions environmental pollution, global surveillance, rapid diagnostics, new vaccines, market entry rewards and innovation funding. There is discussion on investment options and the strategies required for building political consensus around these. The United Kingdom and Chinese governments have each agreed to contribute 50 million GBP (72 million USD) to a Global Innovation Fund, but this requires international support, continued search for further resources and methods for coordinating funding streams (Department of Health [DOH] and UK Government, 2015).

Governmental incentives for public/private partnerships to promote antibiotic research could engender financial support for small drug companies to develop new vaccines and antibiotics (Sukkar, 2013). Smaller businesses developing novel agents may only need a relatively small amount of financial support from their respective governments in order to continue research whilst maintaining viable business. If companies engaging in antibiotic research were exempt from certain tax laws, this might provide enough financial incentive for small to medium-sized firms. Larger companies, pharmaceutical or otherwise, could potentially offer investment and/or financial support to smaller drug companies to aid innovative research (Theuretzbacher, 2012).

Increasing mortality rates due to infection caused by resistant pathogens might confer serious economic consequences to society from healthcare costs and diminishing tax contributions. This hypothetical future scenario can be modeled against the funds required to help develop novel drugs in order to offer justification for investors and ruling bodies. What would public reaction be to an ‘antimicrobial tax,’ for example, and would this be ethically acceptable? Since this tax is aimed at public protection, most people would probably support endorsement, provided the gains receive judicial protection. Imposition of such a tax could hardly be regarded as unethical. This assumes, however, that the tax is directed toward antimicrobial drug discovery and not side-tracked elsewhere.

Pharmaceutical companies themselves have a duty to provide for their employees and that means attention to profit margins in order to stay viable. One innovative project is the Health Impact Fund (HIF), designed to incentivize neglected drug development (Brogan and Mossialos, 2013). The HIF seeks to establish a global repository that will register new medicines and reward companies according to the burden of disease that each drug alleviates (measured in QALY’s). In exchange for reward payments during the first 10 years, companies may then manufacture and distribute the drug at cost over this period. After this, companies would allow generic manufacturing of the drug (Hollis and Pogge, 2008).

Financial incentives could be structured to protect human antibiotics for non-therapeutic purposes, such as growth promotion in animal husbandry and aquaculture. A tax or complete ban should target non-human use of antibiotics that pose a risk of cross-species resistance. The magnitude of this tax would make it economically unattractive to use antibiotics for growth promotion (So and Shah, 2014). There has also been a move toward imposing an ‘antibiotic tax’ on countries with a global wealth above a certain level (Vågsholm and Höjgård, 2010). This would prompt richer countries to control antibiotic consumption, as well as provide support, aid and public services for poorer countries where AMR is more likely to develop and spread. This is an enormous undertaking and vulnerable to fraudulent or corrupt governments that may not correctly, or fairly, distribute financial or other aid from wealthier countries. Perhaps a better strategy would be a drug discovery platform committed to sharing risks; resources; and rewards (Aiello et al., 2006; So and Shah, 2014). Those joining this online community would commit to a clickwrap license not to take from the research commons, nor to privatize the product of their work (So and Shah, 2014). Alternatively, the Access to Medicines Index is a non-profit organization that aims to improve access to medicine in low- and middle-income countries. It bench marks the top 10 R&D companies and highlights current methods undertaken by leading pharmaceutical companies to address access to drugs in poor countries.

## Education, Public Awareness, and Media Campiagns

Septic patients usually receive empirical broad-spectrum agents before microbiological confirmation. Despite advances in molecular testing, microbiology laboratories rely upon overnight culture to characterize pathogens and this can delay targeted clinical assessment. Doctors should review their patients’ results and adjust therapy if necessary, including dose and length of course. If they lack the requisite training to make these decisions, then there is a risk that unsuitable antibiotics are given ‘just in case.’ Current academic training may not adequately embrace non-maleficence regarding antimicrobial therapy, and indeed, basic microbiology teaching itself leaves much to be desired. Many doctors lack sufficient knowledge needed for sensible antimicrobial management (Davenport et al., 2005).

Given the importance of AMR, education on hygiene, infection control, microbiology and prescribing need more focus within universities, hospitals and colleges, ideally supported by hygiene teaching in schools (Lecky et al., 2011). Such a decision rests with governing boards responsible for a particular institute unless national organizations impose specific recommendations. This introduces an important ethical consideration for bodies such as the GMC, Departments of Health and Education, Royal Colleges, veterinary sciences and, arguably, medical and nursing defense unions (Torjesen, 2016).

Of course, it is not just prescribers would benefit from such education. Since AMR affects everyone, there is a strong ethical argument for integrated teaching for all (McNulty et al., 2012). Ordinary people could contribute a significant amount toward combating the spread of infection, and indeed, there is a degree of moral responsibility for them to do so (Curtis et al., 2011). Appropriate hygienic practices for food preparation or visiting the bathroom should be common place, underpinned by education and safeguards for those not able to look after themselves. People should clean their hands after contact with surfaces likely to host pathogens, whether they are multiply resistant or not. Publicizing awareness of AMR and infection prevention will help GPs and pharmacists explain antibiotic use to frustrated and uninformed customers, who fail to realize the consequences from taking antimicrobial drugs. The public do not always know that antibiotics fail to relieve viral infections; or that they should not share medication with another; or hoard or dispose of antibiotics themselves (Jamhour et al., 2017). An appropriately targeted awareness campaign is justifiable because it might influence socially conscious citizens (O’Neill, 2014).

Individuals may not be quite so altruistic when faced with life-threatening infection, however. Richard Lehman tells us that if he acquires a resistant pathogen in hospital, he expects any, or all, of the remaining antimicrobial agents available; regardless of any stewardship policies (Lehman, 2016). Others demand antimicrobial drugs for infected pets, whether or not they are reserved for humans. This behavior demonstrates an economic phenomenon known as the “tragedy of the commons” in which individuals engage in behavior that benefits them at the expense of communal interests (Hardin, 1968). A particular challenge in reducing antibiotic use is this disconnect between individual behavior and population level resistance. Avoiding overuse of a global common good requires making a diffuse and unrecognized social cost visible and felt (Gould and Lawes, 2016).

## Infection Prevention and Control

It is worth considering the courses of action available to society without antibiotics (Dancer, 2013b). These center upon infection prevention and control, which create their own additional ethical considerations (Littmann et al., 2015). Microbial pathogens are spread in a number of ways, usually through physical or respiratory transmission and ingestion. Germs can be acquired merely by touching contaminated surfaces, especially when organisms display environmental longevity. Survival aids transfer of organisms to the next item handled, until natural depletion, demise, or removal through appropriate hygienic practices. High-risk sites are handles, taps, electronic devices, buttons and switches, etc. (Rheinbaben et al., 2000).

Some pathogens become airborne and as such can be directly inhaled or acquired after settling on surfaces. If a man with a cold coughs or sneezes into his hand, then grabs a handrail, he has both released a pathogen into the air, and inoculated the handrail, where it may be acquired by people in close proximity. Airborne dispersal encourages rapid spread of microbes, especially in crowded areas. These facts begin to assign ethical responsibility to the individual, to behave in a hygienic fashion for self-protection, if not to spare other people (Curtis et al., 2011). Sick folk should minimize the risk of transmission to others by taking appropriate action (stay home; cough etiquette; hand hygiene; cover wounds; safe disposal of waste; cleaning; laundry; etc.). Hygienic behavior, however, remains at the mercy of personal choice even if it favors the individual as well as others (Curtis et al., 2011). It would be difficult to enforce appropriate behavior without privacy impositions or, indeed, 24-h surveillance, both of which are markedly more unethical than the behavior they are meant to deter. Pathogens are microscopic, which means that the immediate outcome of unhygienic practices can only be surmised rather than proven.

Raw meat, vegetables, and other foods may be contaminated by pathogens at the point of sale (Fox et al., 2017). If there is insufficient washing or cooking of produce, then organisms are ingested that both infect, and/or colonize, for later complications. Even non-infective microbes pose a risk from contaminated foods, because they may harbor AMRs, which the consumer could retain for years to come. This compromises future management of infection for that individual. The gastrointestinal tract offers an ideal environment for microbial gene exchange (Smillie et al., 2011). Herein, is specific moral responsibility for food suppliers and distributors to manage contamination of foodstuffs at every point in the food chain. The same applies for dirty water, although water quality usually depends upon existing public health and sanitation structures (Lyimo et al., 2016).

Pathogen transmission is especially prevalent in hospitals, due to the close proximity of compromised patients. Hospital-acquired infections (HAIs) can seriously harm patients, prolong hospital stay and initiate costly outbreaks. Many hospitals implement preventive measures to control HAIs, such as hand hygiene, patient screening, isolation, barrier methods, ventilation, and cleaning practices. Isolating patients, while useful for containing the spread of infection, brings its own ethical considerations (Abad et al., 2010). Screening patients has also been subjected to ethical debate, especially when it constitutes an invasive assault on a patient (e.g., genital swabbing) (Millar, 2009). Why inflict such a procedure on a patient if it is not going to benefit that patient? The patient at risk of MDRO carriage may not necessarily care if he or she shares his or her untreatable parasite with other people. While doctors are obliged to practice beneficence, patient behavior remains exempt from similar restriction.

Most infection prevention activities lack hard evidence except for hand hygiene and cleaning. Cleaners do not always clean thoroughly, however, and clinical staff do not always wash their hands. This has encouraged a range of monitoring strategies along with threats (‘zero tolerance’) to improve and maintain hygiene standards (Dancer, 2010). Thus, ‘stewardship’ for infection prevention and control revolves around monitoring and feedback, which challenges wellbeing and even employment prospects for staff (Dancer, 2010). Hand hygiene itself, with repeated reminders, threats and audits, can cause the avid practitioner much grief with sore or blistered hands. Cleaning staff are expected to use powerful disinfectant chemicals for decontaminating the hospital environment; these, also, can cause personal discomfort, for both respiratory system and skin, whether or not they have been appropriately managed (Zock et al., 2010).

Further concerns have been raised over microbiocidal products used in healthcare following confirmed links between disinfectants and resistance (Sattar, 2010). There has even been a call for regulatory use (Kampf, 2016). Powerful disinfectants harm the surface ecology, much like antibiotics harm healthy gut flora, permitting naturally tolerant or resistant microflora to survive and create reservoirs of increasingly resistant microbes (Dancer, 2013a). People at home or hospital staff may do more harm than good by routinely using these products; and toxicity and cost offer further challenges. Ethical arguments exist over routine exposure of environments to potent disinfectants, given that links have been demonstrated between these chemicals and MDROs. The FDA has recently issued a mandate to disinfectant companies to justify the components of their produce or remove them from sale (US Food and Drug Administration [FDA], 2016).

Ultimately, responsibilities for infection control fall to everyone, whether they are healthcare staff or not. However, there should be a balance between specific preventive interventions and the damage they may do to budgets, environment and human well-being. In the past, people with untreatable infections such as syphilis and tuberculosis were subjected to a range of public health interventions including imprisonment in order to curtail spread of disease (Kousoulis et al., 2011; Abrams, 2013). Public health bodies may be faced with similar ethical dilemmas this century (Littmann et al., 2015).

## Alternative Therapies

There is a range of alternative anti-infectives, both ancient and modern. These include ‘old fashioned’ remedies such as cranberry juice; honey; maggots; salt; heavy metals such as silver and copper, sunlight and fresh air; homeopathy; plants, herbs, and spices; or more recently, electrolysed water; UV irradiation; antimicrobial peptides; phage therapy; immunological strategies and nanotechnology (Dancer, 2013b). There is a risk that desperate folk seeking therapy for untreatable infection might experiment with untried, untested, and highly dubious therapies. These alternative solutions also dictate their own ethical debate over efficacy, toxicity, and cost. Few have been subjected to blinded randomized trials and therefore remain controversial. Indeed, setting up a double blind randomized clinical trial on exposure to fresh air, for example, would pose considerable challenge, despite nursing patients with tuberculosis outdoors just a few decades ago. Since there are ongoing studies on many of these alternative therapies, it appears that concern over AMR has tipped equipoise so that studies on these ‘old wives’ remedies are now regarded as legitimate ethical enquiry.

Doctors can circumvent prohibited use of novel drugs by following established guidance on unconventional prescribing (Montanaro et al., 2016). Some remedies already mentioned are not medicines, but foodstuffs, natural products or irrigants. There are no formal policies for use, although it is well-recognized within the medical profession that ‘compassionate’ use of an unlicensed product is permissible, provided the prescriber and patient have considered the evidence, discussed all options and agreed on management. Such prescribing should always be performed openly, with appropriate documentation. Perhaps novel products that do show promise from compassionate use will spur researchers to set up formative clinical trials and thus potentially benefit everyone (Raus, 2016).

## Conclusion

Many of the activities described in this article would forestall the spread of AMR to a greater or lesser extent, provided they are implemented and upheld by the correct authorities. While there is no question over the need to institute control initiatives now and for the future, we should remember that biologic antimicrobials are ultimately doomed to fail. It is happening now, and will continue to happen, due to widespread use and selection pressure from antimicrobial agents. AMR is a simple illustration of Darwinian evolution, in that any drug, which impedes a biological process (without killing the organism), will initiate a microbial mechanism to resist the insult (Rodríguez-Rojas et al., 2013). Microbes, like man, are programmed to survive.

While this article has attempted to balance the principles of bioethics against antimicrobial stewardship, it has uncovered a complex minefield. Do policy makers have the right to gamble upon; dictate; or even enforce, current antimicrobial choice? If effective therapy is urgently required for an individual, or outbreak situation, then should specific populations be denied helpful and even potentially lifesaving drugs (Littmann and Viens, 2015)? A septic patient should receive best guess empirical therapy; without condemnation of either prescriber or recipient. Perhaps we should try harder to improve current prescribing practices, regardless of what may or may not happen in the future. Indeed, traditional understanding of bioethical principles contemplates only the here and now; prescribing amoxicillin to a patient with suspected urinary tract infection, ticks all the boxes for beneficence without due recognition of the future consequences for that patient, others, and environment. Maybe that explains why current measures remain undefined, insufficient and subject to prescriber whim. There is evidence from the consumption rates in hospitals and community to demonstrate that antibiotics are still being overprescribed, and these data do not include over-the-counter or internet sources (Millar, 2012).

If humanity only has the privilege of antibiotics for a short time, there should be more effort toward conserving drugs before populations are devastated by untreatable infection (Littmann et al., 2015). Multi-drug resistant tuberculosis has swept across countries that lack public health infrastructure, now delivering a global threat courtesy of travel and migration. India is home to the world’s largest epidemic of MDRTB, which affects 2 million people every year, with a death roughly every 2 min (Overdorf, 2015). Some strains are resistant to all previously active antibiotics, thus rendering them completely untreatable. It cannot be a coincidence that the ‘White Plague,’ as TB was termed a century ago, originally stimulated the search for a ‘magic bullet’ for infection. Now MDRTB requires immediate global initiative to fund yet more research and development into finding alternative therapies.

Controlling AMR does not have one single solution but there are strategies that have immediate effect. Firstly, infection prevention and control (Aiello et al., 2006; Dancer, 2013b). If infections are adequately contained, then horizontal transmission of multi-resistant bacteria will be decreased. While individuals have a moral responsibility for personal hygiene, multiple organizations clearly have judicial responsibility toward infection control. It is not difficult to embed a framework for infection prevention into healthcare, public buildings, schools, colleges, industry, food establishments, and even private households (Bloomfield, 2015). Hand hygiene and cleaning provide simple yet fundamental practices for breaking the chain of infection (Curtis et al., 2011). Contractual cleaning specifications are a lot easier to implement than activities that rely upon behavioral decisions, i.e., hand hygiene, even if the latter is underpinned by ethical education or conscience.

Secondly, much more can be done toward stewardship practices in hospitals, community and indeed, worldwide. There are still too many antimicrobial agents used inappropriately and unnecessarily, and mandatory restrictions are probably just around the corner. A crack down on internet, black market, and over-the-counter sales ought to receive immediate attention. There may also be a call for implementing an antimicrobial prescribing license for all prescribers, underpinned by prioritizing basic education on microbiology and anti-infectives.

Thirdly, more incentives are required for companies and academic institutions to initiate research into new drugs and vaccines, including novel methods for managing infection. Granting or investing in financial support for small drug companies and businesses to aid research allows early exploration of compounds and vaccines discovered by scientists and doctors. One of these could result in the single most important medical and scientific breakthrough of the 21st century. Approval agencies need to accelerate new medicines, balanced against regulatory hurdles, while conserving safety and efficacy standards.

Fourthly, there is need for greater international collaboration and accountability distribution, with broader engagement of countries and United Nations agencies to foster global intersectoral action on AMR (Jasovský et al., 2016). This includes agreed definitions for surveillance and support for analyses and reporting of trends on a worldwide basis. Control of disease and its causes is impossible without surveillance and international liaison. More understanding of the economic analyses which help drive health behaviors on antimicrobial use would benefit policy makers and stewardship strategies (Eggleston et al., 2010).

Finally, it is time for a widespread public awareness campaign in order to educate everyone about the potential loss of anti-infective drugs (Micallef et al., 2017). Social media and television reach far more people than notices pinned up in doctors’ clinics. Although there is no better public awareness than a fatal outbreak of multi-resistant bacteria (or a celebrity succumbing to untreatable infection), governments and societies should educate the public about AMR now and how it affects them and future generations. Making it personal will challenge ignorance. For prescribers themselves, discussion on the traditional definitions of bioethical principles might contribute toward a deeper understanding of AMR and provide the support required to modify prescribing behavior on a global basis.

## Author Contributions

BP, PH, and LK conceived the idea of examining the ethics of controlling antimicrobial resistance and produced an initial draft under the supervision of RB; NW updated and revised the section on the veterinary and agricultural aspects of AMR and SD revised and rewrote the manuscript, adding in material on hospital and community prescribing and infection control. All authors have seen and approved the final draft.

## Conflict of Interest Statement

The authors declare that the research was conducted in the absence of any commercial or financial relationships that could be construed as a potential conflict of interest.
